# The role of education as a socialization mechanism in addressing the social gradient in depression treatment in Belgium (2004–2018)

**DOI:** 10.3389/fsoc.2025.1204794

**Published:** 2025-03-04

**Authors:** Lisa Colman, Katrijn Delaruelle, Piet Bracke

**Affiliations:** Department of Sociology, HeDeRa (Health and Demographic Research), Ghent University, Ghent, Belgium

**Keywords:** depression, depression treatment, mental health care use, income inequalities, educational inequalities

## Abstract

**Introduction:**

Previous studies have identified socioeconomic inequalities in the treatment of depression. However, these studies often take a narrow approach, focusing on a single treatment type and lacking a comprehensive theoretical framework. Moreover, income and education are frequently used interchangeably as indicators of disadvantage, without distinguishing their unique impacts. This study argues that relying solely on income to explain treatment inequalities is overly simplistic, suggesting instead that education influences treatment through two distinct pathways. The study’s objectives are twofold: first, to investigate the presence of a social gradient in depression treatment, and second, to examine how this gradient is manifested.

**Methods:**

This study utilizes data from the Belgian Health Interview Survey (BHIS), covering four successive waves: 2004, 2008, 2013, and 2018. The weighted data represent a sample of the adult Belgian population. Multinomial regression models are used to address the research aims, and models are plotted to detect trends over time using marginal means post-estimation.

**Results:**

Findings indicate that income is not significantly related to depression treatment, while persistent educational inequalities in treatment are observed over time. Individuals with longer educational attainment are more likely to use psychotherapy alone or a combination treatment, whereas individuals with shorter educational attainment are more likely to use pharmaceutical treatment alone.

**Discussion:**

This study demonstrates that education plays a critical role in fostering health-related knowledge and reasoning, making individuals with longer education more likely to engage in rational health behaviors and choose more effective treatments, even when these treatments require more effort and competencies. The findings underscore the importance of considering education as a key determinant of depression treatment inequalities.

## Introduction

### Depression prevalence and treatment

Depression, characterized by persistent sadness and lack of interest or pleasure, affects individuals worldwide (Marcus et al., 2012). It encompasses emotional, cognitive, and somatic symptoms and significantly impairs functioning. Data from 2018 reveal that nearly 1 in 10 Belgians experienced depressive symptoms, with more than half meeting criteria for severe depression (Gisle et al., 2018). Depression prevalence is subject to disparities influenced primarily by socioeconomic factors (World Health Organization, 2014). In Belgium, individuals in disadvantaged socioeconomic positions report depressive symptoms more frequently than those in more advantaged positions (Gisle et al., 2018), aligning with prior research that indicates a social gradient in depression prevalence (Hirschfeld and Cross, 1982; Kessler and Bromet, 2013; Lorant et al., 2003; Lund et al., 2010; Perry, 1996).

The primary treatments for depression include psychotherapy and pharmaceutical interventions (Karyotaki et al., 2014). These approaches stem from distinct theoretical perspectives on the origins of depression, with the biomedical perspective historically dominating mental health care. However, recognition of psychotherapy’s importance in treating depression has grown (Cuijpers et al., 2020; Kamenov et al., 2017; Spijker et al., 2013). In 2014, the Belgian Health Care Knowledge Centre conducted a meta-analysis of international scientific literature on the effectiveness of psychotherapy, concluding that psychotherapy alone or combined with antidepressants is preferable depending on depression severity (Karyotaki et al., 2014; Karyotaki et al., 2016). For severe depression, combination treatment is recommended (Cuijpers et al., 2020; Davey and Chanen, 2016; Driessen et al., 2020; van Bronswijk et al., 2019).

### Inequalities in depression treatment

Research indicates that socioeconomic position is linked not only to depression prevalence but also to disparities in depression treatment. However, existing studies primarily focus on pharmaceutical interventions. For instance, uninsured individuals in the United States, often from lower-income groups, are less likely to receive antidepressant treatments (Olfson and Marcus, 2009). Conversely, studies from Denmark show that lower-income individuals have a higher prevalence of antidepressant treatment (Andersen et al., 2009; Hansen et al., 2004). Similarly, Finnish research indicates that individuals with shorter educational backgrounds are more likely to access older antidepressants with more side effects, while those with longer education are more likely to receive newer antidepressant medications (Halonen et al., 2018).

Disparities in psychotherapy treatment are less explored. Research indicates that individuals with shorter educational attainment have a reduced probability of receiving outpatient psychotherapy in Germany (Albani et al., 2010). Similar associations between shorter education and decreased access to psychotherapy have been observed in studies from France and Finland (Briffault et al., 2008; Suoyrjö et al., 2007).

However, these studies lack a comparative analysis of the associations between socioeconomic position and various types of depression treatment. Addressing disparities in one form of treatment necessitates considering inequalities in others. Furthermore, these studies are often descriptive and lack strong theoretical foundations. Socioeconomic indicators, such as income and education, are frequently used without adequately distinguishing their roles in generating and sustaining social inequality. Therefore, the objective of this study is to examine the existence of a social gradient in the choice of depression treatment and to analyze the ways in which this gradient becomes evident.

## Theoretical approaches

Inequalities in depression treatment from an income-related perspective often focus on individuals’ financial resources (Mojtabai, 2009; Veugelers and Yip, 2003). Limited financial means may hinder access to costly mental health services, leading to restricted treatment options (Loef et al., 2021; Veugelers and Yip, 2003). Although treatment costs may play a role, attributing treatment inequalities solely to income is overly simplistic.

To gain a more comprehensive understanding of treatment inequalities, the role of education must also be considered. Over recent decades, education has emerged as a key socioeconomic indicator in health research for several reasons (Shavers, 2007; Winkleby et al., 1992). Unlike income, education is typically established during early adulthood and maintains a high level of stability. It is usually acquired earlier in the life course and partially contributes to an individual’s income level. Furthermore, the causal relationship between education and health is less susceptible to reverse causality compared to the link between income and health. Empirical evidence consistently points to education as the most potent and reliable socioeconomic predictor of health outcomes.

Two pathways highlight education’s influence on treatment considerations. One pathway emphasizes education as a mechanism of allocation within the labor market (Mirowsky and Ross, 2005), where longer education is linked to improved employment prospects and conditions. This mechanism has intensified alongside the expansion of education and the increasing credentialization of society (Baker, 2014; Baker, 2011; Schofer and Meyer, 2005). Better job prospects and working conditions, in turn, shape the decision-making process regarding treatment choices.

The second pathway emphasizes education as a socialization mechanism. Here, education itself plays a significant role in shaping treatment choices. Socialization mechanisms are central to the theory of learned effectiveness (Mirowsky and Ross, 2005), which represents the health sociological interpretation of the human capital approach proposed by Becker (1964). According to this perspective, education fosters the development of intangible resources, such as knowledge, social competence, and “lifestyles” (Mirowsky and Ross, 2005), that could potentially influence treatment decisions. Furthermore, a cumulative effect may occur when individuals with longer educational trajectories find themselves in occupations that further enhance their higher levels of effectiveness.

Both pathways assume a causal link between education and health; however, spurious factors can also influence this relationship. The connection between education and health may be driven by shared characteristics that impact both educational attainment and health outcomes (Cutler and Lleras-Muney, 2006; Montez and Friedman, 2015). For example, “social background” is an influential factor, with higher socioeconomic position correlating with educational attainment (Kloosterman et al., 2009; Müller and Karle, 1993; Pfeffer, 2008) as well as better health outcomes (Kestilä et al., 2006). Nonetheless, amidst these confounding influences, a portion of the education-health association is likely causal.

The fundamental cause theory suggests that socialization mechanisms contribute to treatment inequalities (Link and Phelan, 1995, 2010). Individuals with longer education levels are better able to choose effective treatments, irrespective of costs. Additionally, diffusion of innovation theory posits that educated individuals are more likely to adopt new and effective therapies, which may further contribute to inequalities (Rogers, 2003).

However, the consideration extends beyond the mere effectiveness of treatments; the complexity of treatments also holds significance (Link and Phelan, 1995, 2010). Certain treatments require sustained effort and complex actions, and individuals with advanced education tend to be more inclined to undertake treatments that require greater commitment (e.g., long-term psychotherapy) and competencies (psychotherapy is linguistic and demands sustained effort). Moreover, the rise of new therapies initially leads to an increase in inequality, which may later decrease. The more complex the new therapy, the greater its impact on inequality (Clouston and Link, 2021).

Health capital approaches to treatment inequalities align with the hierarchical relationships assumed by the aforementioned theories. However, they introduce two-way interactions within the diffusion process of innovations, such as new therapies. These approaches recognize that resources for adopting innovations are derived from past experiences and habitual actions (Shim, 2010). The concept of “cultural health capital” resembles self-efficacy and health literacy, adding to the complexity narrative. Health-literate individuals, particularly in psychotherapy, facilitate effective communication and tailored treatment, equipping them with resources that provide material benefits in care. Health care providers may perceive these skills as indicators of motivation and competence, potentially leading to more favorable responses. Such skills can initiate sequences of interactions, including improved information sharing and comprehensive responses to enhance communication and care.

In summary, while the fundamental cause theory and diffusion research emphasize resources and agency, structural influences on diffusion are also crucial (Freese and Lutfey, 2011). Recognizing cultural health capital, individuals are not merely strategic agents but are also shaped by habitus. In this view, the use of health-promoting resources, like depression treatments, is not always exclusively purpose-driven. Instead, the accumulation and mobilization of resources result from past experiences and deeply ingrained, habitual ways of thinking and organizing actions (Clouston and Link, 2021).

### Belgium’s institutional context

As highlighted by Freese and Lutfey (2011), the role of structural constraints in influencing treatment diffusion is crucial. Therefore, inequalities in depression treatment cannot be fully understood without considering the institutional context. An example is Belgium’s mental health care organization and reimbursement structure, which has traditionally been characterized by a biomedical orientation. Until 2016, psychiatrists were the only recognized specialists in mental health (Kohn et al., 2016). Individuals in Belgium seeking help for mental health concerns were largely unaware of alternative mental health professionals outside of general practitioners (GPs) and psychiatrists. Although clinical psychologists received official recognition in September 2016, legal provisions for psychotherapy reimbursement were lacking, and only limited reimbursement was offered by various health insurance funds, contingent on specific conditions (Ector, 2016). More recent policy changes, enacted in March 2019 and September 2021, allowed the Belgian Health Insurance to begin partially reimbursing consultations with private psychologists (Mistiaen et al., 2019; Van Maldegem, 2021).

In many countries, systems are in place to ensure affordable access to psychotherapy, often through professional associations or cooperatives with a social focus. In Belgium, however, access to affordable psychotherapy has historically been limited. Additional support structures exist, such as increased allowances for lower-income individuals, which provide higher reimbursement rates, and community health centers, which offer accessible mental health care within certain regions (Rijksinstituut voor Ziekte-en Invaliditeitsverzekering, n.d.). Nevertheless, coverage remains partial, and recent reimbursement policies for private psychologists aim to bridge this gap further (Mistiaen et al., 2019; Van Maldegem, 2021). In contrast, considerable funds are allocated for the provision of affordable antidepressants. Data on medication spending indicate that antidepressants constitute a significant portion of mental health expenditures in Belgium (Casteels et al., 2010), reflecting the greater reimbursement coverage by the Belgian Health Insurance for pharmaceutical treatments (Mistiaen et al., 2019).

However, an increasing focus on pro-therapeutic policies has emerged to address the high prescription rates and widespread use of pharmaceutical treatments. Over the past decade, Belgian research has consistently underscored the significant role of GPs in prescribing antidepressants (Boffin et al., 2012; Fraeyman et al., 2012). In response, measures have been implemented to raise GPs’ awareness of their prescribing practices, including updated guidelines for adult depression treatment (Vandekerckhove, 2016), aimed at reducing antidepressant prescriptions (Declercq et al., 2017).

Moreover, GPs may exhibit varied approaches in treating individuals with depression, influenced by patients’ socioeconomic position (Balsa and McGuire, 2003; Hyde et al., 2005; Willems et al., 2005). Qualitative research, for instance, reveals that GPs frequently adapt their clinical management strategies based on patients’ socioeconomic circumstances, aiming to provide care that is cost-effective, practical, and attainable (Bernheim et al., 2008; Hyde et al., 2005). Disparities in GP-patient interactions have also emerged, with patients in more disadvantaged socioeconomic positions being more likely to receive pharmaceutical treatments. This reflects perceptions that these patients possess not only limited financial resources but also diminished social-cognitive capacities for engaging in more “active treatments” (Butterworth et al., 2013; Fraeyman et al., 2012). Previous research has also suggested that differential patient preferences, reinforced by GPs, may contribute to treatment disparities. For instance, one study hypothesized that patients with higher levels of education are more assertive in seeking non-pharmacological treatment options and that GPs are more likely to support these preferences (Hyde et al., 2005).

### Hypotheses

Based on the aforementioned theoretical approaches, we propose the existence of a social gradient in depression treatment. Specifically, we hypothesize that individuals with shorter educational attainment are more likely to opt solely for pharmaceutical treatment and less likely to choose psychotherapy or a combination treatment. This trend is expected to be only partially attributable to their lower income level. Conversely, we hypothesize that individuals with longer educational attainment are more inclined to select either psychotherapy alone or a combination treatment, with a lower likelihood of opting for pharmaceutical treatment alone, independent of their income level. Recognizing the evolving nature of this issue, the study incorporates time trends to analyze changes in depression treatment patterns and associated inequalities over time.

## Methods

### Sample

Data were obtained from the Belgian Health Interview Survey (BHIS), a repeated cross-sectional survey coordinated by Sciensano. Four successive waves are included: 2004, 2008, 2013, and 2018. Households and their members were selected from the National Register using a multi-stage stratified sampling procedure. Information was collected through face-to-face interviews and a self-administered questionnaire. This study includes 2,298 Belgian respondents over the age of 25 who reported depression complaints in the 12 months prior to data collection. Details on respondent selection criteria and data cleaning procedures are provided in Appendix 1.

### Variables

Appendix 2 contains a table presenting univariate statistics for the variables used.

The dependent variable assesses the type of treatment respondents pursued in the context of self-reported depression complaints over the past 12 months. This variable includes four categories: 1 = pharmaceutical treatment (use of antidepressants for depression in the past 12 months), 2 = psychotherapy treatment (use of psychotherapy for depression in the past 12 months), 3 = combination treatment (use of both antidepressants and psychotherapy for depression in the past 12 months), and 4 = no treatment.

The independent variable, education, is assessed based on the highest level of education achieved and classified into three groups according to the International Standard Classification of Education (ISCED) of 2011 (UNESCO, 2012): 1 = shorter education (pre-primary or primary education), 2 = intermediate education (lower-and upper-secondary education), and 3 = longer education (post-secondary or tertiary education) [ref. cat.].^1^

The mediator variable, household income, is presented in quintiles to reflect income distributions within Belgium. This variable includes four categories: 1 = low household income (1st and 2nd quintiles) [ref. cat.], 2 = median household income (3rd and 4th quintiles), 3 = high household income (5th quintile), and 4 = missing data. Missing data are included as a separate category due to 12.3% missing responses.

Several covariates are also included, such as gender, age, nationality, urbanization, region, survey wave, GP contact in the past 12 months, regular GP status, frequency of social contact, and household composition. Gender (0 = female) and nationality (0 = Belgian) are binary variables. Age is grouped into three categories: 1 = 25–44 years [ref. cat.], 2 = 45–64 years, and 3 = 65+ years. Region is categorized as 1 = Flanders [ref. cat.], 2 = Brussels, and 3 = Wallonia. Urbanization levels are divided into 1 = cities/agglomerates [ref. cat.], 2 = urban/suburban, and 3 = rural. Wave is represented as a categorical variable: 1 = 2004 [ref. cat.], 2 = 2008, 3 = 2013, and 4 = 2018. GP contact in the past 12 months (0 = no) and regular GP status (0 = no) are binary variables. Frequency of social contact has three categories: 1 = less than once a week [ref. cat.], 2 = more than once a week, and 3 = missing data. Household composition is classified as 1 = single or single-parent household [ref. cat.], 2 = couple with or without children, and 3 = other household composition.

### Statistical procedure

Prevalence rates of depression treatment are reported using weighted proportions and stratified by education, household income, and survey wave. Following bivariate statistical analyses, two multinomial regression models are tested, estimating relative risk ratios (RRRs) and their corresponding *p*-values. In the first model, education and the covariates are included; in the second model, both education and household income are included along with the covariates. Both multinomial regression models compare each treatment category against the reference category—pharmaceutical treatment, which is the most commonly followed treatment. For each of the two models, treatment categories are also plotted (using marginal means post-estimation) to detect trends over time. Analyses are weighted to adjust for survey sampling and non-participation bias and are conducted using SPSS 28 and STATA 15.

## Results

Bivariate results (see Table 1) reveal that, across waves, the predominant treatment choice remains pharmaceutical treatment alone (52.0%), followed by combination treatment (25.5%) and psychotherapy alone (4.8%). Additionally, a notable portion of individuals opts for no treatment (17.6%). Educational disparities in treatment preferences are evident: individuals with shorter educational attainment (65.6%) tend to favor pharmaceutical treatment more than those with intermediate (47.4%) or longer education (37.0%). Conversely, individuals with longer education show a greater preference for psychotherapy (10.6%) and combination treatment (34.2%). Similar but less pronounced patterns are observed across household income groups. Individuals with lower income (52.9%) are more likely to choose pharmaceutical treatment than those with medium (44.9%) or higher income (41.5%). Additionally, those with higher income (12.5%) show a greater inclination toward psychotherapy. However, the impact of income differences on combination treatment is modest.

**Table 1:** Weighted proportions^a^ of treatment for self-reported depression in the past 12 months, stratified by wave, education and household income^c^.

	Pharmaceutical	Psychotherapy	Combination	No treatment	*p*-values^b^
	%	%	%	%	
**2004–2018** (*N* = 2,298)	52.05 (*N* = 1,196)	4.83 (*N* = 111)	25.54 (*N* = 587)	17.58 (*N* = 404)	
Education					0.0000***
Shorter education	65.60	1.10	13.99	19.30	
Intermediate education	47.39	4.98	28.83	18.81	
Longer education	37.00	10.60	34.20	18.20	
Household income					0.0000***
Low income	52.87	3.20	25.52	18.41	
Mediate income	44.91	6.41	27.99	20.69	
High income	41.48	12.51	30.19	15.81	
**2004** (*N* = 580)	75.17 (*N* = 436)	1.72 (*N* = 10)	10.00 (*N* = 58)	13.10 (*N* = 76)	
Education					0.2717
Shorter education	79.88	0.00	7.79	12.32	
Intermediate education	69.01	2.26	16.12	12.61	
Longer education	63.79	2.25	21.21	12.75	
Household income					0.0011***
Low income	71.42	1.73	17.34	9.51	
Mediate income	81.24	0.88	8.22	9.65	
High income	50.65	2.98	28.84	17.53	
**2008** (*N* = 429)	49.88 (*N* = 214)	3.73 (*N* = 16)	32.63 (*N* = 140)	13.75 (*N* = 59)	
Education					0.0100*
Shorter education	64.24	1.93	16.58	17.25	
Intermediate education	46.68	4.66	39.63	9.03	
Longer education	31.50	5.94	49.16	13.40	
Household income					0.7329
Low income	45.85	1.80	39.54	12.81	
Mediate income	44.01	4.13	39.17	12.69	
High income	52.64	10.30	32.10	4.96	
**2013** (*N* = 598)	47.66 (*N* = 285)	4.18 (*N* = 25)	27.59 (*N* = 165)	20.57 (*N* = 123)	
Education					0.0002***
Shorter education	62.31	1.39	9.83	26.47	
Intermediate education	48.63	3.79	24.07	23.51	
Longer education	37.96	12.50	33.79	15.74	
Household income					0.0074**
Low income	54.61	3.11	19.18	32.1	
Mediate income	43.12	5.04	23.76	28.08	
High income	47.96	17.24	26.55	8.24	
**2018** (*N* = 691)	37.77 (*N* = 261)	8.68 (*N* = 60)	32.42 (*N* = 224)	21.13 (*N* = 146)	
Education					0.0142*
Shorter education	53.44	1.44	27.03	18.09	
Intermediate education	35.96	7.52	33.22	23.30	
Longer education	25.98	15.30	34.54	24.17	
Household income					0.3874
Low income	40.30	5.25	30.99	23.46	
Mediate income	30.02	11.09	35.74	23.15	
High income	29.05	15.21	31.90	23.84	

a Crude rates, unadjusted for other indicators.

b Weighted Pearson chi-square test for the entire studied period and per wave.

c Percentages on missing category of household income not shown in table.

Over time, the exclusive use of pharmaceutical treatment has consistently declined (2004 = 75.2%; 2018 = 37.8%), while the adoption of combination (2004 = 10.0%; 2018 = 23.3%) and psychotherapy treatments (2004 = 1.7%; 2018 = 8.7%) has increased. However, even in the latest wave, psychotherapy alone remains relatively low. The proportion of individuals not seeking treatment has also grown over time (2004 = 13.1%; 2018 = 21.1%). Educational and income disparities in treatment persist across waves.

Multinomial regression results from the first model (see Table 2) show a significant association between education and depression treatment choices. Specifically, individuals with shorter (RRR = 0.18, *p* < 0.01) or intermediate education (RRR = 0.44, *p* < 0.01) are less likely than those with longer education to choose psychotherapy over pharmaceutical treatment. This pattern is also observed in combination treatment (shorter education = 0.38, *p* < 0.001; intermediate education = 0.70, *p* < 0.05). No significant educational differences are evident for individuals who opt not to seek treatment (shorter education = 0.70, *p* > 0.05; intermediate education = 0.79, *p* > 0.05).

**Table 2:** Weighted RRRs and *p*-values of treatment for self-reported depression in the past 12 months by education, household income, and covariates.

		Model 1				Model 2		
	(Base^a^)	Psychotherapy	Combination	No treatment	(Base)	Psychotherapy	Combination	No treatment
		RRR - *p* > |*z*|	RRR - *p* > |*z*|	RRR - *p* > |*z*|		RRR - *p* > |*z*|	RRR - *p* > |*z*|	RRR - *p* > |*z*|
**Education** (ref. cat.: longer education)								
Intermediate education		0.44**	0.70*	0.79		0.50*	0.68*	0.79
Shorter education		0.18**	0.38***	0.70		0.21**	0.36***	0.70
**Household income** (ref. cat.: low income)								
Mediate income						1.38	0.91	1.18
High income						2.13	0.81	0.96
(Missings)						1.33	0.76	0.94
Gender (ref. cat.: female)		1.40	0.88	0.99		1.44	0.87	0.98
Age (ref. cat.: 25–44)								
45–64		0.43**	1.02	0.81		0.43**	1.03	0.81
65+		0.06***	0.25***	0.84		0.07**	0.25***	0.85
Nationality (ref. cat.: non-Belgian)		0.99	0.57*	1.87*		1.05	0.56*	1.92**
Urbanization (ref. cat.: cities-agglomerates)								
Suburban–urban		1.57	1.30	1.00		1.53	1.29	0.99
Rural		1.17	0.91	0.70		1.20	0.91	0.71
Region (ref. cat.: Flanders)								
Brussels		1.60	1.54*	0.69		1.63	1.53*	0.70
Wallonia		0.64	1.13	0.66*		067	1.12	0.66*
Wave (ref. cat.: 2004)								
2008		5.18**	4.08***	1.42		5.09**	4.17***	1.43
2013		5.87**	2.33**	2.54***		5.86**	2.33**	2.50***
2018		12.64***	4.50***	3.54***		12.08***	4.55***	3.50***
GP contact past 12 months (ref. cat.: no)		0.46	0.67	0.25		0.45	0.67	0.25***
Regular GP (ref. cat.: no)		0.45	1.99	0.57		0.42	2.00	0.57
Social contact (ref. cat.: less than once a week)								
More than once a week		0.93	0.93	1.03		0.90	0.93	1.03
(Missings)		0.44	1.08	0.90		0.43	1.10	0.90
Household composition (ref. cat.: single/one parent household)								
Couple with/without children		1.18	0.87	1.04		0.95	0.91	1.01
Another household composition		1.74	0.90	1.04		1.54	0.93	0.99
Intercept		0.27	0.28	1.91		0.23	0.30	1.84

a The reference category (base) for the multinomial regression is pharmaceutical treatment.

*p-value < 0.05; **p-value < 0.01; ***p-value < 0.001.

In the second model, which includes both education and household income (see Table 2), education continues to show a significant association with depression treatment. These findings align with those of the first model. However, income does not exhibit a significant association with treatment choice. Additionally, the results indicate that older adults are less likely to favor psychotherapy (ages 45–64: 0.43, *p* < 0.01; ages 65+: 0.07, *p* < 0.01) over pharmaceutical treatment. Individuals aged 65 and older are also less inclined to choose combination treatment (0.25, *p* < 0.001). Non-Belgian respondents show a reduced likelihood of choosing combination treatment (0.56, *p* < 0.05) and are more likely to opt for no treatment (1.92, *p* < 0.01) compared to pharmaceutical treatment. Regional differences are also apparent. Individuals from Brussels are more likely to choose combination treatment (1.53, *p* < 0.05) than those from Flanders, while individuals from Wallonia are less likely to abstain from treatment (0.66, *p* < 0.05) than their Flemish counterparts. Over time, the likelihood of individuals choosing psychotherapy (2008 = 5.09, *p* < 0.01; 2013 = 5.86, *p* < 0.01; 2018 = 12.08, *p* < 0.001) and combination treatment (2008 = 4.17, *p* < 0.001; 2013 = 2.33, *p* < 0.01; 2018 = 4.55, *p* < 0.001) has increased relative to pharmaceutical treatment. However, in the last two waves, there is also an increased likelihood of individuals not pursuing treatment (2013 = 2.50, *p* < 0.001; 2018 = 3.50, *p* < 0.001). Lastly, individuals who had contact with a GP in the past 12 months are less likely to abstain from treatment (0.25, *p* < 0.001).

**Figure 1:**
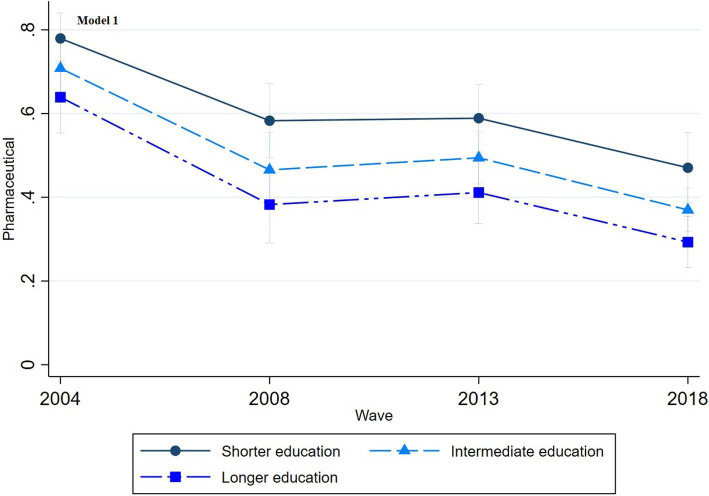
Trends in pharmaceutical treatment by education across the waves (2004-2018), corresponding to Model 1 in Table 2.

**Figure 2:**
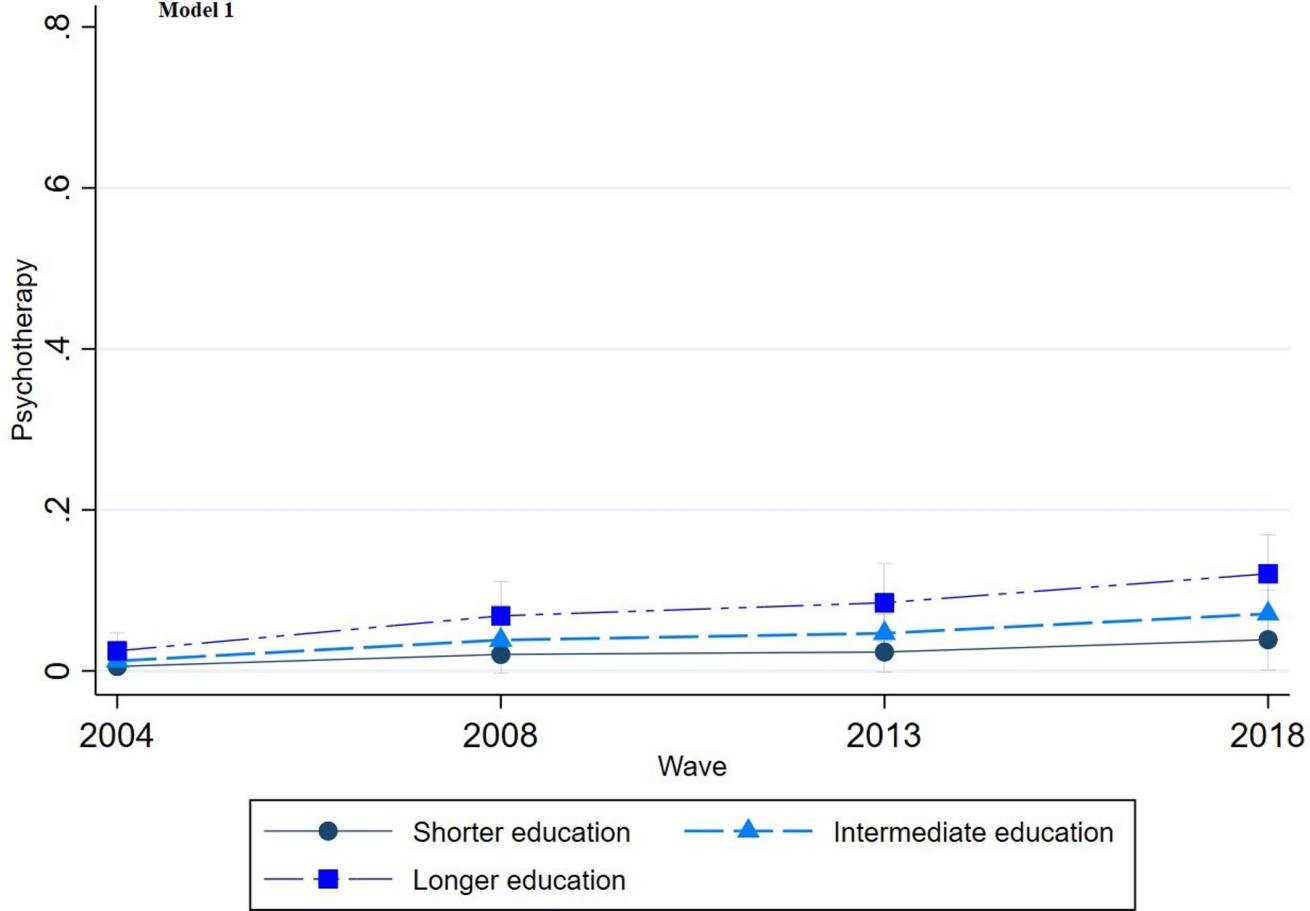
Trends in psychotherapy treatment by education across the waves (2004-2018), corresponding to Model 1 in Table 2.

**Figure 3:**
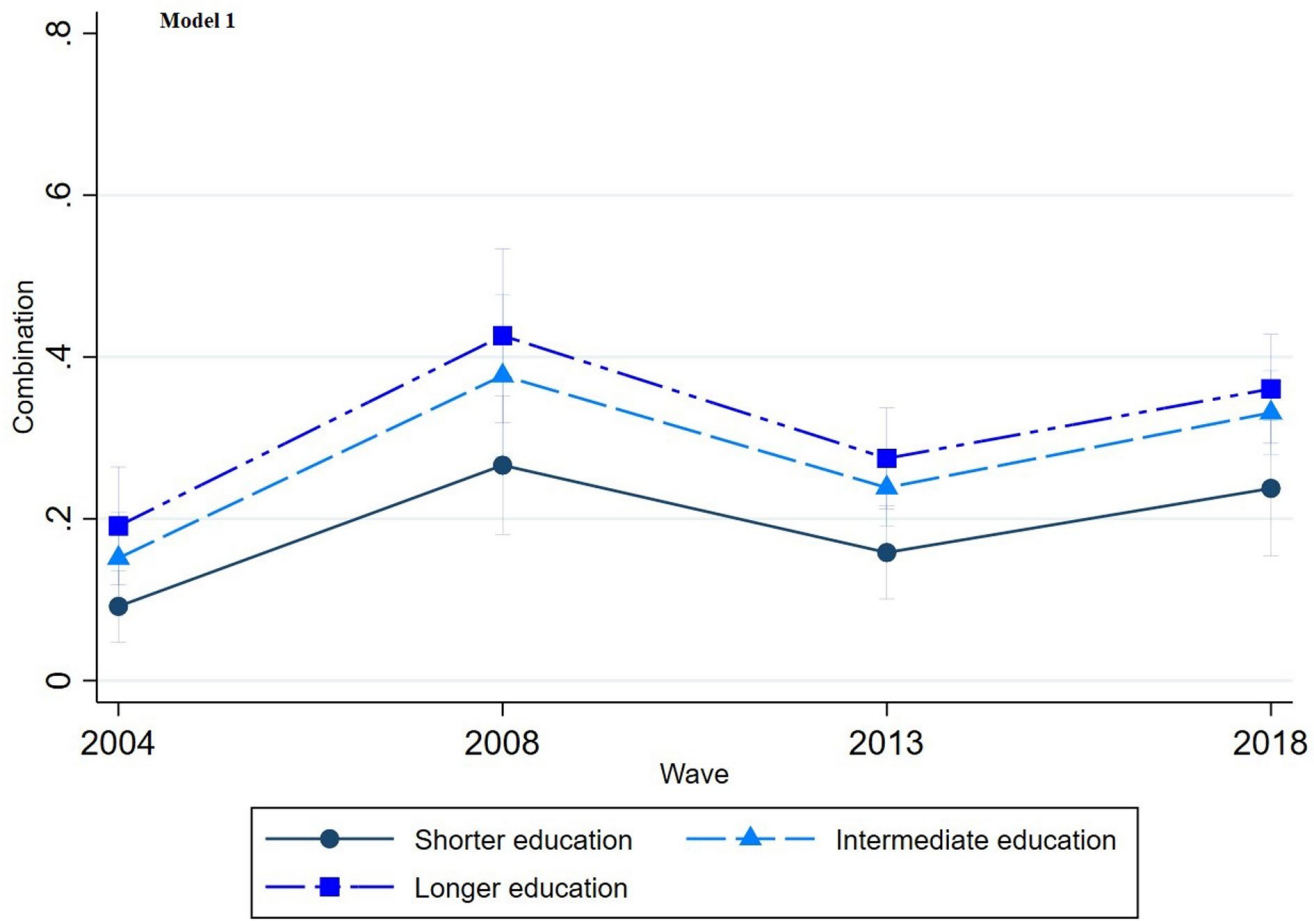
Trends in combination treatment by education across the waves (2004-2018), corresponding to Model 1 in Table 2.

**Figure 4:**
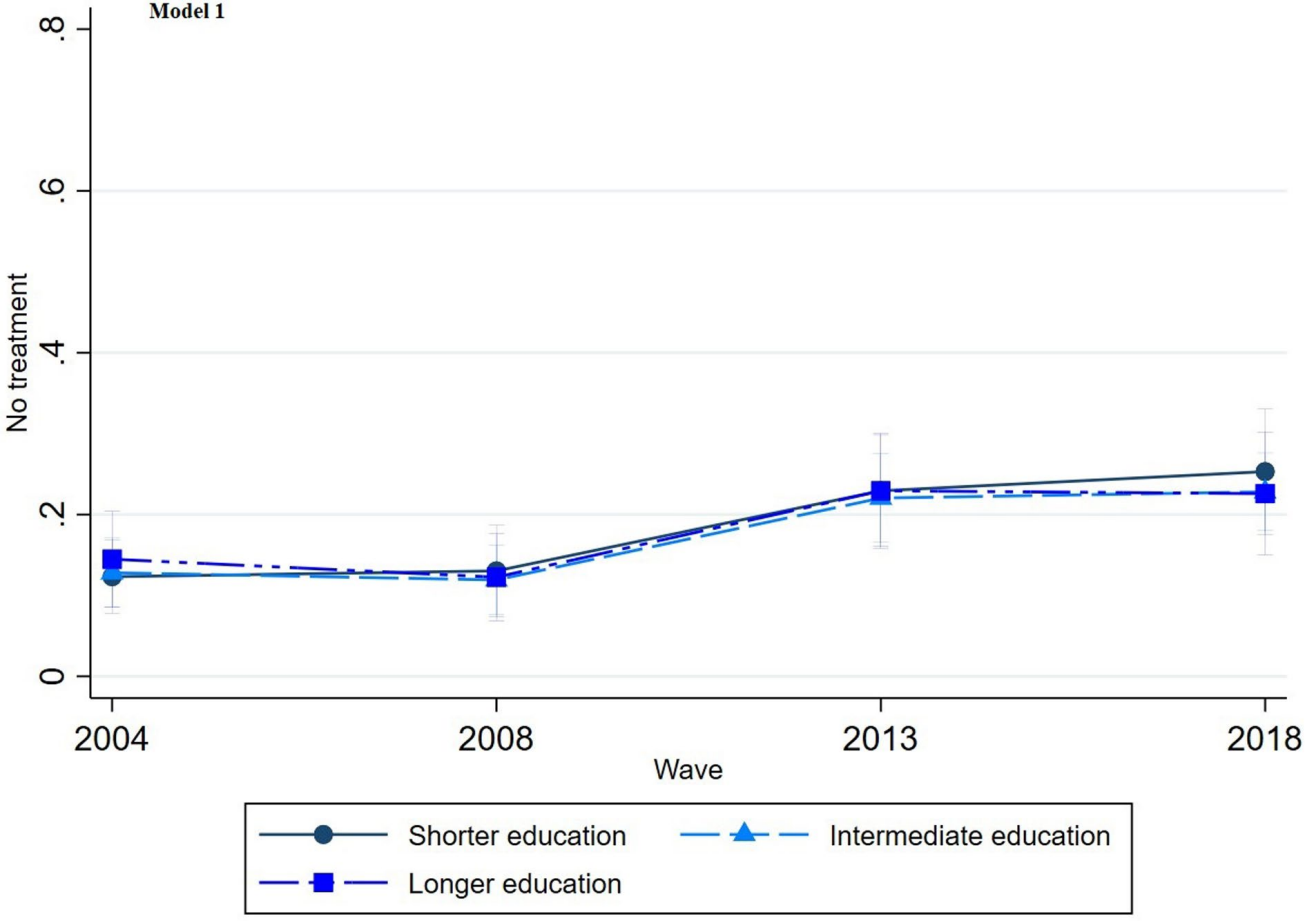
Trends in no treatment by education across the waves (2004-2018), corresponding to Model 1 in Table 2.

**Figure 5:**
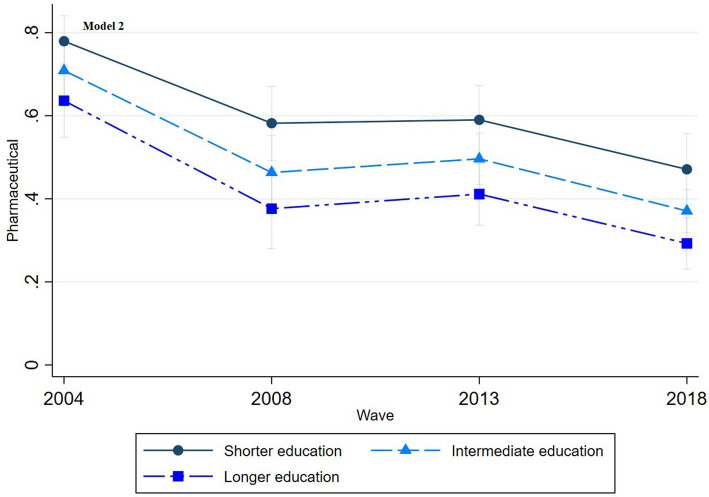
Trends in pharmaceutical treatment by education across the waves (2004-2018), corresponding to Model 2 in Table 2.

**Figure 6:**
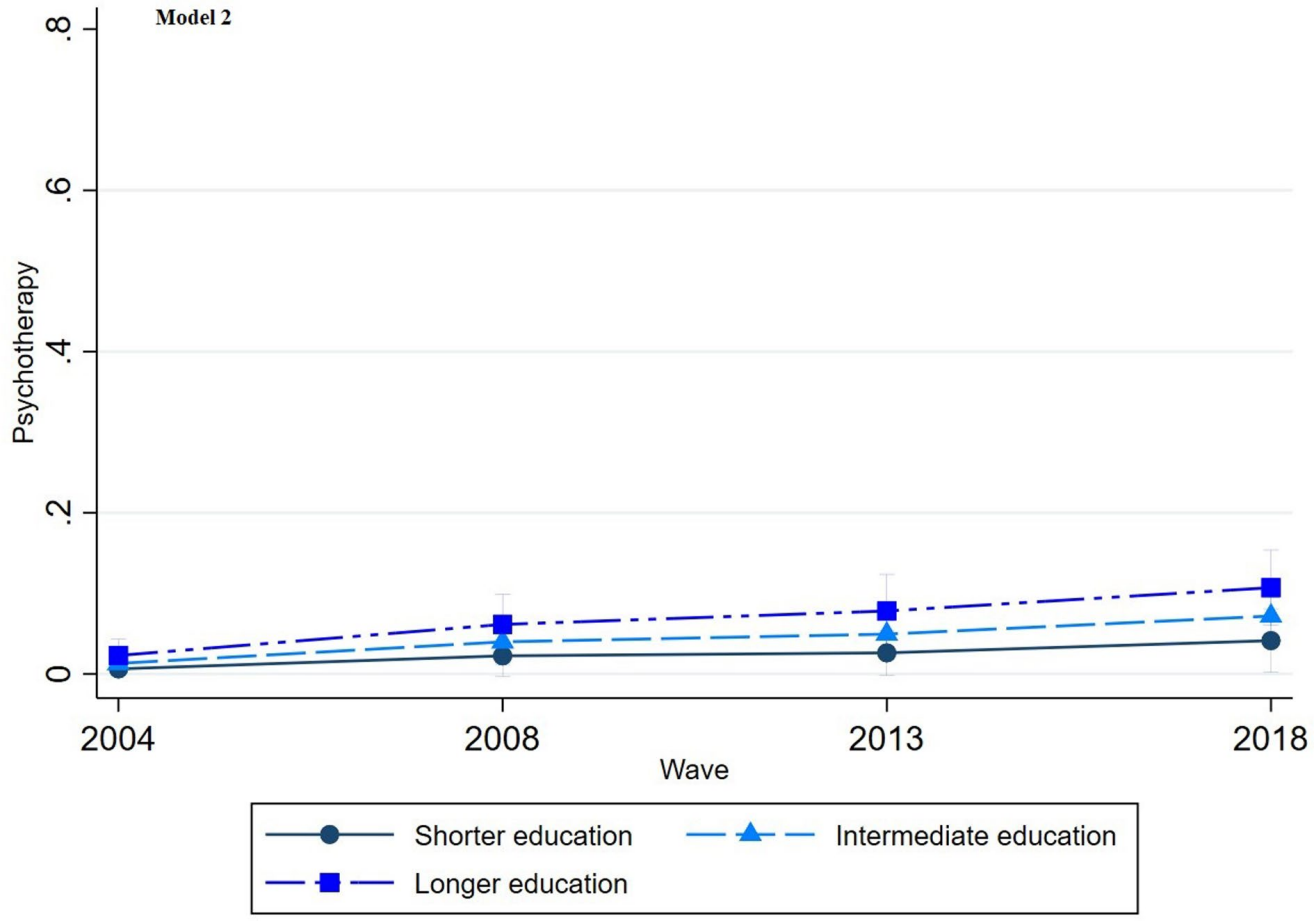
Trends in psychotherapy treatment by education across the waves (2004-2018), corresponding to Model 2 in Table 2.

**Figure 7:**
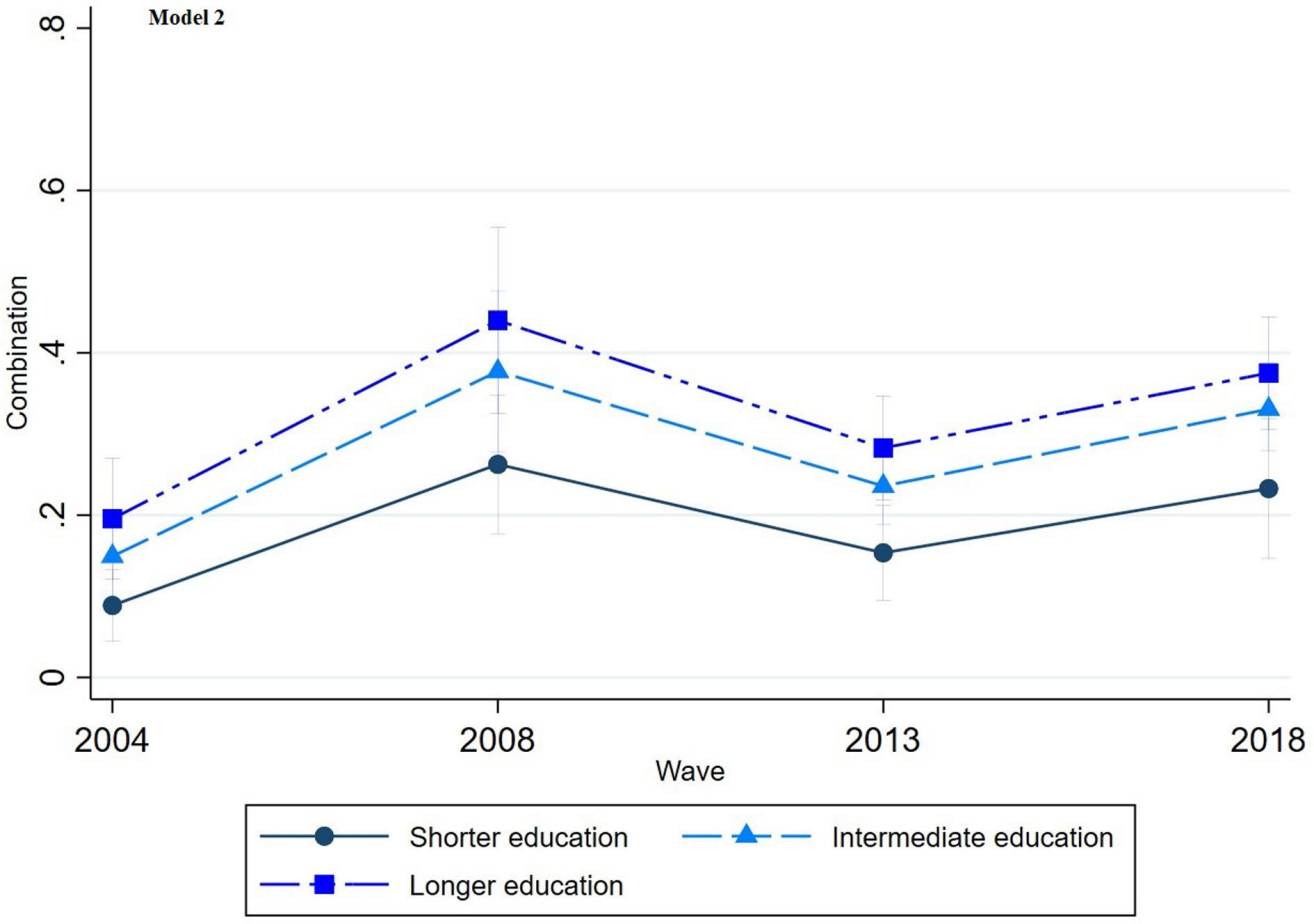
Trends in combination treatment by education across the waves (2004-2018), corresponding to Model 2 in Table 2.

**Figure 8:**
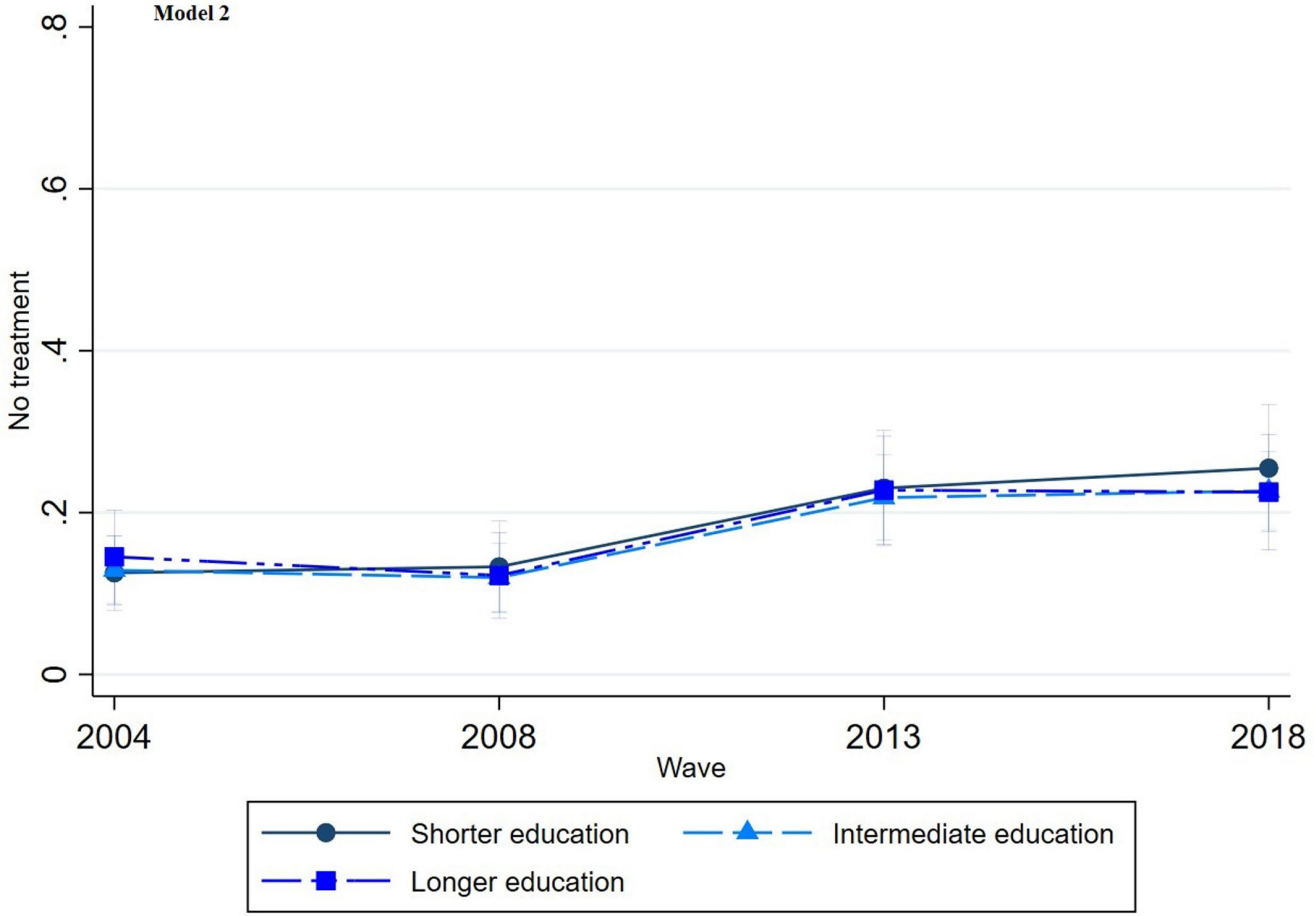
Trends in no treatment by education across the waves (2004-2018), corresponding to Model 2 in Table 2.

The multinomial regression graphs (see Figures 1–8) provide further insights into temporal trends and model-specific findings. Overall, the graphs show a consistent decline in the use of pharmaceutical treatment, accompanied by an increase in psychotherapy and combination treatment usage. The prevalence of individuals not seeking treatment has also risen over time. The graphs from the first model highlight persistent educational disparities across waves. Meanwhile, the graphs from the second model demonstrate that the educational gradient remains significant even after accounting for household income, suggesting that income does not mitigate the observed educational differences.

## Discussion

Our study reveals several important findings. First, household income does not appear to be related to depression treatment, while education clearly influences depression treatment, with significant and persistent differences observed over time. Consistent with our hypothesis, we found that individuals with longer educational attainment are more likely to choose psychotherapy or combination treatment, irrespective of their income. Conversely, individuals with shorter educational attainment are more likely to rely on pharmaceutical treatment alone, even though this approach is considered less optimal and effective (Cuijpers et al., 2020).

Attributing “health benefits” solely to financial resources is therefore insufficient. Supporting both the theory of learned effectiveness (Mirowsky and Ross, 2003) and the concept of cultural health capital (Shim, 2010)—which emphasize the human capital component of education—it can be argued that education fosters health-related knowledge, logic, and competencies. Thus, individuals with longer education are more inclined to engage in effective, rational health behaviors and possess better skills for selecting appropriate treatments (Lawrence, 2017). Additionally, they are more likely to invest in treatments requiring significant effort, skills, and competencies, underscoring education’s importance in shaping health-related decisions and behaviors (Ross and Mirowsky, 2000; Shim, 2010). This aligns with findings from a Canadian study, which also emphasizes the primacy of education among socioeconomic factors that enable effective mental health care use (Steele et al., 2007). Consistent with our findings, this study similarly concluded that income level did not independently relate to mental health care use.

Regarding important temporal trends, the exclusive use of pharmaceutical treatment for depression has decreased, while the adoption of psychotherapy and combination treatments has increased. This shift corresponds with Belgian guidelines (Karyotaki et al., 2014; Superior Health Council, 2019) as well as international recommendations (Jobst et al., 2016; World Health Organization, 2016), which increasingly advocate for the inclusion of psychotherapy as part of depression treatment, either alone or in combination with antidepressants. Antidepressant monotherapy alone is no longer regarded as optimal care for depression (Karyotaki et al., 2014). The decline in sole pharmaceutical treatment may also be due to growing awareness of the side effects and withdrawal challenges associated with long-term antidepressant use (Guy et al., 2020). Studies have shown that combined treatment involving psychotherapy has better acceptance rates, resulting in lower dropout rates and a higher likelihood of recovery (Cuijpers et al., 2014; De Jonghe et al., 2001; Ormel et al., 2022). A meta-analysis by Kamenov et al. (2017) also concluded that combined psychotherapy and pharmaceutical treatments perform significantly better than either treatment alone. Both psychotherapy and pharmaceutical treatments are effective for improving functioning; however, when adjusted for publication bias, psychotherapy was found to be more efficacious than pharmacotherapy.

The adoption of “new practices,” such as new depression treatments, initially tends to generate inequalities, with disproportionate use among individuals with longer education (Mackenbach, 2012). This finding aligns with fundamental cause theory (Link and Phelan, 1995; Link and Phelan, 2000) and the broader diffusion of innovations literature (Rogers, 2003). Socialization mechanisms suggest that adopting new practices is not simply about having the financial resources to enable adoption but also the competencies to understand and apply these practices effectively (Lawrence, 2017). This proficiency is shaped by habitual ways of thinking and organizing actions, described as “habitus” (Shim, 2010).

For example, a slight increase in inequality in the use of psychotherapy alone is visible in the graphs. This trend may reflect patient empowerment among individuals with longer education. The traditional model, where GPs make treatment decisions on behalf of patients, is gradually being replaced by one in which patients actively participate in their treatment choices (Camacho, 2014), such as expressing a preference for non-pharmaceutical options. A study of Houle et al. (2013) also found that individuals with a university degree are more inclined to opt for psychotherapy than those with shorter education. With increased awareness of potential side effects and withdrawal challenges, individuals with longer education are better equipped to communicate effectively during psychotherapy and more willing to invest the energy and time required, underscoring attributes more common among those with more formal education.

Some nuances in interpreting the psychotherapy findings are also important. The relatively low number of individuals using psychotherapy alone may be influenced by the significant role of GPs in Belgium’s mental health care system (Boffin et al., 2012; Fraeyman et al., 2012). It is likely that most respondents who reported experiencing depression within the past 12 months consulted a GP, as GPs serve as the primary care contact. Since GPs are the main prescribers of antidepressants, it can be assumed that many patients received antidepressant-based treatment. A study also revealed that, despite abundant mental health resources in Belgium, referral rates to mental health specialists remain low (Kovess-Masfety et al., 2007). A recent qualitative study in Belgium observed that many GPs even permit patients to request repeat antidepressant prescriptions without an appointment, reflecting challenges GPs face in altering routines and instituting regular, proactive reviews of antidepressant prescriptions (Van Leeuwen et al., 2021).

Combination treatment is disproportionately less used among individuals with shorter education, likely influenced by GPs’ treatment approaches. Studies have shown that GPs often tailor treatment decisions based on patients’ socioeconomic position (Bernheim et al., 2008; Hyde et al., 2005). For instance, research has found that GPs perceive individuals with shorter education as lacking the resources to manage more “active” treatments. A Norwegian study supporting this notion observed that patients with shorter education levels receive shorter consultations but undergo more medical tests per visit (Brekke et al., 2018). The quality of these consultations correlates with patients’ communicative or cognitive proficiencies, more often associated with education than income. This finding suggests that GPs may be more likely to endorse a “medical approach” when treating individuals with shorter education levels.

## Research implications

Institutional factors such as treatment policies, reimbursement regulations, and the role of GPs can significantly influence disparities in depression treatment. To gain a comprehensive understanding of how these institutional factors contribute to inequalities, future studies should use nuanced measures of institutional variables, such as first-or second-line treatment prescriptions, frequency of GP interactions, and psychotherapy waiting lists in specific regions. Another recommendation is to use dimensional indicators instead of categorical ones to independently measure “need” (mental health status) and “treatment,” as suggested by Coghill and Sonuga-Barke (2012), to allow a more nuanced analysis of treatment disparities. Longitudinal studies that follow individuals over time could also provide valuable insights into how changes in socioeconomic position, such as income, influence shifts in treatment choice, offering a dynamic understanding of treatment disparities.

Considering the growing trend toward non-pharmaceutical mental health approaches, future research in Belgium could examine recent psychotherapy reimbursement regulations, introduced in March 2019 and September 2021, which aim to improve accessibility and affordability (Mistiaen et al., 2019). However, our findings suggest that these regulatory changes may not suffice to eliminate social disparities in the utilization of effective treatments.

## Limitations

This study has two important limitations. First, the dependent variable “depression treatment” was measured only for respondents who self-reported experiencing depression within the past 12 months, which significantly reduced the initial sample. This decision was made by Sciensano (BHIS coordinator). Second, self-reported data can be influenced by respondents’ individual perceptions (Jylhä, 2009). While previous studies have supported the validity and reliability of self-reported health information (Halford et al., 2012; Pu et al., 2013; Santos et al., 2021), the stigma surrounding depression may contribute to self-report bias (Chan and Mak, 2017; Hunt et al., 2003).

## Conclusion

This study presents two primary conclusions. First, it identifies a distinct social gradient in depression treatment, with education significantly shaping treatment decisions. Individuals with longer education are more likely to choose psychotherapy or combination treatment, while those with shorter education are more inclined toward pharmaceutical treatment alone. This discrepancy persists over time, underscoring education’s persistent influence. Second, the findings emphasize the inadequacy of attributing “health benefits” solely to financial resources; instead, education plays a critical role in guiding rational health behaviors and treatment decisions. The trend toward integrating psychotherapy, often combined with antidepressants, also reflects a shift away from antidepressant monotherapy. The evolving role of patient agency in treatment choices is also highlighted, as patients’ active participation in their treatment decisions grows in importance. These findings underscore the complex interplay between education, patient empowerment, and the evolving landscape of depression treatment.

## Data Availability

The datasets presented in this article are not readily available because the data will not be deposited since its subject to a contract with Sciensano (The Scientific Institute of public health of the federal Belgian State). Requests to access the datasets should be directed to https://www.sciensano.be/nl/node/55737/gezondheidsenquete-aanvraagprocedure-microgegevens.

## Footnotes

^1^Reference category.
