# Supplementary material for: The role of education as a socialization mechanism in addressing the social gradient in depression treatment in Belgium (2004–2018)
Source: Front Sociol. 2025 Mar 4;10:1204794. doi: 10.3389/fsoc.2025.1204794 (PMC11914119; doi:10.3389/fsoc.2025.1204794)
Supplement: Supplementary file 1 [file Data_Sheet_1.pdf]

Supplementary Material

The role of education as a socialization mechanism in addressing the social gradient in depression treatment in Belgium (2004-2018)

L. Colman*, K. Delaruelle, P. Bracke

*** Correspondence:** Corresponding author: Lisa.Colman@UGent.be

Supplementary Data

Appendix 1. Respondent selection criteria and data cleaning.

Respondents who self-reported experiencing depression within the twelve months preceding data collection were included in the sample (N=4,106). Self-reported depression indicates that no specific diagnostic tools were used to assess depressive symptoms; rather, respondents themselves reported a history of depression. These respondents were then asked about their use of antidepressants and psychotherapy in the past twelve months, which served as the basis for the dependent variable, "depression treatment," categorized into four groups: 1=pharmaceutical treatment, 2=psychotherapy treatment, 3=combination treatment, and 4=no treatment. Cases with missing values for the dependent variable were excluded, yielding a sample of 2,580 respondents. Next, respondents under the age of twenty-five, as well as those with missing values for the independent variable or other relevant variables (when missing values accounted for less than 5% of the data), were also excluded. This selection process resulted in a final sample of 2,298 respondents.

Appendix 2. Univariate statistics for the variables used (N = 2,298).

	N (%)		N (%)
**Wave**		**Nationality**	
2004	580 (25.24)	Belgian	2,039 (88.73)
2008	429 (18.67)	Non-Belgian	259 (11.27)
2013	598 (26.02)	**Urbanisation**	
2018	691 (30.07)	Cities-agglomerates	1,153 (50.17)
**Treatment**		Suburban-urban	793 (34.51)

Pharmaceutical treatment	1,196 (52.05)	Rural	352 (15.32)
Psychotherapy treatment	111 (4.83)	**Region**	
Combination treatment	587 (25.54)	Flanders	694 (30.20)
No treatment	404 (17.58)	Brussels	684 (29.77)
**Education**		Wallonia	920 (40.03)
Longer education	546 (23.76)	**GP contact past 12 months**	
Intermediate education	1,212 (52.74)	No	108 (4.70)
Shorter education	540 (23.50)	Yes	2,190 (95.30)
**Household income**		**Regular GP**	
High income	1,032 (44.91)	No	68 (2.96)
Mediate income	754 (32.81)	Yes	2,230 (97.04)
Low income	229 (9.97)	**Social contact**	
(Missings)	283 (12.32)	Less than once a week	475 (20.67)
**Gender**		More than once a week	1,269 (55.22)
Male	733 (31.90)	(Missings)	554 (24.11)
Female	1,565 (68.10)	**Household composition**	
**Age**		Single/one parent household	1,157 (50.35)
25-44	650 (28.29)	Couple with/without children	963 (41.91)
45-64	988 (42.99)	Another household composition	178 (7.75)
65+	660 (28.72)		
